# *JAK2^V617F^* Drives Mcl-1 Expression and Sensitizes Hematologic Cell Lines to Dual Inhibition of JAK2 and Bcl-xL

**DOI:** 10.1371/journal.pone.0114363

**Published:** 2015-03-17

**Authors:** Jun Guo, Lisa Roberts, Zhui Chen, Philip J. Merta, Keith B. Glaser, O. Jameel Shah

**Affiliations:** Oncology Division, Research & Development, AbbVie Inc., North Chicago, Illinois, United States of America; Emory University, UNITED STATES

## Abstract

Constitutive activation of the Janus kinase (JAK)/signal transducer and activator of transcription (STAT) axis is fundamental to the molecular pathogenesis of a host of hematological disorders, including acute leukemias and myeloproliferative neoplasms (MPN). We demonstrate here that the major *JAK2* mutation observed in these diseases (*JAK2^V617F^*) enforces Mcl-1 transcription via STAT3 signaling. Targeting this lesion with JAK inhibitor I (JAKi-I) attenuates STAT3 binding to the Mcl-1 promoter and suppresses Mcl-1 transcript and protein expression. The neutralization of Mcl-1 in *JAK2^V617F^*-harboring myelodyssplastic syndrome cell lines sensitizes them to apoptosis induced by the BH3-mimetic and Bcl-xL/Bcl-2 inhibitor, ABT-263. Moreover, simultaneously targeting JAK and Bcl-xL/-2 is synergistic in the presence of the *JAK2^V617F^* mutation. These findings suggest that JAK/Bcl-xL/-2 inhibitor combination therapy may have applicability in a range of hematological disorders characterized by activating *JAK2* mutations.

## Introduction

Inappropriate STAT activation plays a central role in the molecular pathogenesis of a range of hematologic disorders including acute myeloid leukemia (AML) [1,2], acute lymphoblastic leukemia (ALL) [3,4] and chronic myelogenous leukemia (CML) [5] as well as the myeloproliferative neoplasms polycythemia vera (PV), essential thrombocytopenia (ET), and primary myelofibrosis (PMF). This is generally explained by the high frequency of somatic mutation in genes encoding tyrosine kinases proximal to STAT3/5 such as *FLT3*, *KIT*, *JAK2*, *JAK3*, and *MPL*. Though several oncogenic *JAK2* variants have been described, *JAK2* mutation manifests primarily as a single non-conservative substitution (V617F) in the JH2 pseudokinase domain. This lesion disables the auto-inhibitory interaction between pseudokinase domain and activation loop residues producing a constitutively active kinase. As *JAK2* mutation is observed in nearly all cases of PV, *JAK2* mutational status is now a major diagnostic criterion for this disease. Moreover, *JAK2* or *MPL* mutation in ET and PMF is considered diagnostic of clonal hematopoeisis [6,7], and JAK mutations are found at high frequency in relapsed ALL [8]. Several small-molecule inhibitors of JAK2 are in clinical development for PV, ET, and PMF [9], and Ruxolitinib (formerly INCB18424) has received FDA approval for PMF.

The STAT target genes Mcl-1 and Bcl-XL collaborate to oppose apoptosis mediated by pro-apoptotic BH3-only proteins [10,11]. We reasoned that mutational activation of Jak2 may enforce Mcl-1 and/or Bcl-XL expression, whereas inhibition of JAK2 in this context may reduce the expression of these pro-survival Bcl-2 family members. Expression of Mcl-1 represents a barrier to apoptosis induced by the Bcl-2 family inhibitors, ABT-737 and ABT-263 [10,12, 13], which inhibit Bcl-XL, Bcl-2, and Bcl-w [14,15]. Thus, a reduction in Mcl-1 shifts the burden to maintain cell survival to Bcl-XL, thereby lowering the threshold for apoptosis mediated by Bcl-XL/-2 inhibition. As combination chemotherapy has become a mainstay in clinical oncology, we set out to ascertain the potential utility of combining JAK and Bcl-2 family inhibitors as therapy in *JAK2* ^*V617F*^-positive leukemias.

## Materials and Methods

### Cell Culture and Extraction

JAK inhibitor I (JAKi-I; cat# 420099) was purchased from Calbiochem. SET-2, HEL, MV4;11, and K562 cells were obtained from ATCC and cultured as recommended. UKE-1 cells were purchased from Walter Fiedler (University of Hamburg). Cell lysates were either prepared using CHAPS lysis buffer (10 mM HEPES, pH 7.4, 150 mM NaCl, 1% CHAPS) or cell extraction buffer containing 1% Triton X-100, 0.1% SDS, and 0.5% deoxycholate. All buffers were supplemented with protease and phosphatase inhibitor cocktails prior to use.

### Immunoprecipitation

For immunoprecipitation, lysates were prepared in CHAPS lysis buffer and 2 mg of cell lysate was mixed with at least 8 μg of immunoprecipitating antibody overnight at 4°C. The following day, 30 μl of a Protein A- or Protein G-agarose slurry was added for an additional 2 hr. Immunoprecipitates were washed three times in CHAPS lysis buffer, and heated in 1.5x loading buffer at 95°C for 5 min.

### siRNA Transfection and Cell Viability Assay

Transfection of siRNAs was performed using Lipofectamine RNAiMAX according to the manufacturer’s recommendations. Cell viability was determined using the alamarBlue cell viability assay (Invitrogen) according to manufacturer’s suggested protocol after exposure to drug combinations for 72 hr. Caspase-3 activity was determined using the Caspase-GLO 3/7 Assay (Promega) in parallel with the CellTiter-GLO viability assay (Promega). The data are expressed as Caspase-3/7 activity divided by cell viability.

### TR-FRET and ChIP assays

Ki values of JAKi-I for individual kinases were determined by time-resolved fluorescence resonance energy transfer (TR-FRET) by displacement of proprietary Oregon Green-labelled probes with test compounds. ChIP using STAT3 antibodies was carried out using the EZ-ChIP assay kit (Millipore).

### Statistical Analysis

Synergistic activities of JAKi-I and ABT-263 were determined using the Bliss additivity model [16] where the combined response C of both agents with individual effects A and B is C = A + B—(A▪B) and where A and B represent the fractional inhibition between 0 and 1. Combined response scores greater than 0.15 were considered synergistic and scores lower than-0.15 were considered antagonistic.

## Results

### Regulation of Mcl-1 and Bcl-XL by *JAK2* ^*V617F*^

**Fig 1:**
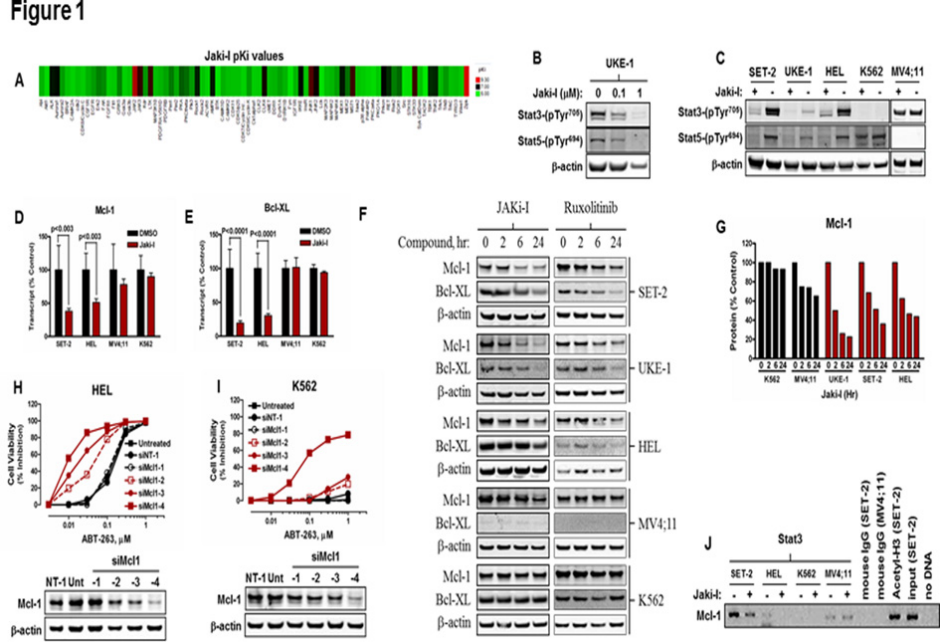
Regulation of Mcl-1 and Bcl-XL by *JAK2* ^*V617F*^. (A) JAKi-I was evaluated in a panel of 66 human protein kinases as detailed in the Methods section, and Ki values determined. Red, <0.01 μM; black, 0.01–1.67 μM, green, >1.67 μM. (B) UKE-1 (*JAK2* ^*V617F*^) AML cells were treated for 10 min with JAKi-I as indicated. Tyrosine phoshorylation of STAT3 and STAT5 was determined by immunoblotting. (C) The *JAK2* ^*V617F*^-positive AML cell lines, SET-2, UKE-1, and HEL, the chronic myelogenous leukemia line, K562 (*JAK2* ^*V617F*^-negative), and the AML cell line, MV4;11 (*JAK2* ^*V617F*^-negative), were cultured in the absence of serum for 2 hr, then treated with 1 μM JAKi-I for 1 hr. Constitutive tyrosine phosphorylation of STAT3 and STAT5 was determined by immunoblotting. (D and E) Cells were treated for 6 hr with JAKi-I, and the abundance of Mcl-1 and Bcl-XL mRNA was determined by qPCR. Data represent means +/- standard deviation for two independent determinations each performed in triplicate. (F) Cells were treated with JAKi-I or Ruxolitinib over a 24-hr time course, and Mcl-1 and Bcl-XL levels were determined by immunoblotting (similar results were observed for 2 separate immuoblots). (G) Quantification of the data shown in (F). Data are expressed as the ratio of intensity of Mcl-1/β-actin for each time point. (H and I) HEL or K562 cells were transfected with either non-targeting (siNT-1) or Mcl-1-specific (siMcl1–1–4) siRNAs, treated for 72 hr with ABT-263, then lysates were prepared, and cell viability was determined. Data are means of duplicate samples and are representative of two independent experiments. (J) Cells were treated for 6 hr with or without 1 μM JAKi-I then subjected chromatin immunoprecipitation assays using normal mouse IgG, anti-acetylated histone H3, or anti-STAT3. Mcl-1 promoter binding was determined by PCR on chromatin immunoprecipitates (for immunoblots, similar results were obtained twice).

JAKi-I is a selective inhibitor of JAK2 (Fig. 1A) and induces the rapid, dose-dependent inhibition of phosphorylation of both STAT3 and STAT5 (Fig. 1B). All leukemia lines tested displayed constitutive phosphorylation of STAT3/5 in the absence of serum, but only in cell lines carrying the *JAK2* ^V617F^ mutation was STAT3/5 phosphorylation inhibited following treatment with JAKi-I (Fig. 1C). Mcl-1 and Bcl-XL transcript and protein levels (Fig. 1D-G) sharply declined over a 24-hr time period following JAK inhibition, and similar results were observed with Ruxolitinib, a clinical relevant drug. Although Mcl-1 protein can also be regulated by protein degradation, protein stability was not altered upon JAKi-I treatment in the presence of cycloheximide (data not shown). Chromatin immunoprecipitation experiments demonstrated that STAT3 interacted with the *MCL1* promoter (Fig. 1J). Promoter binding was disrupted following treatment with JAKi-I in cell lines expressing *JAK2* ^V617F^, but not in cell lines without this lesion.

Reducing the levels of Mcl-1, irrespective of *JAK2* mutation, sensitizes leukemia cells to ABT-263 (Fig. 1H-I), indicating that Bcl-2 family proteins, such as Bcl-xL and Bcl-2, are necessary to maintain viability when Mcl-1 levels are reduced.

### Combination of JAK2 Inhibitor and ABT-263 Yields Synergistic Activity in *JAK2* ^*V617F*^-Harboring AML Cell Lines

**Fig 2:**
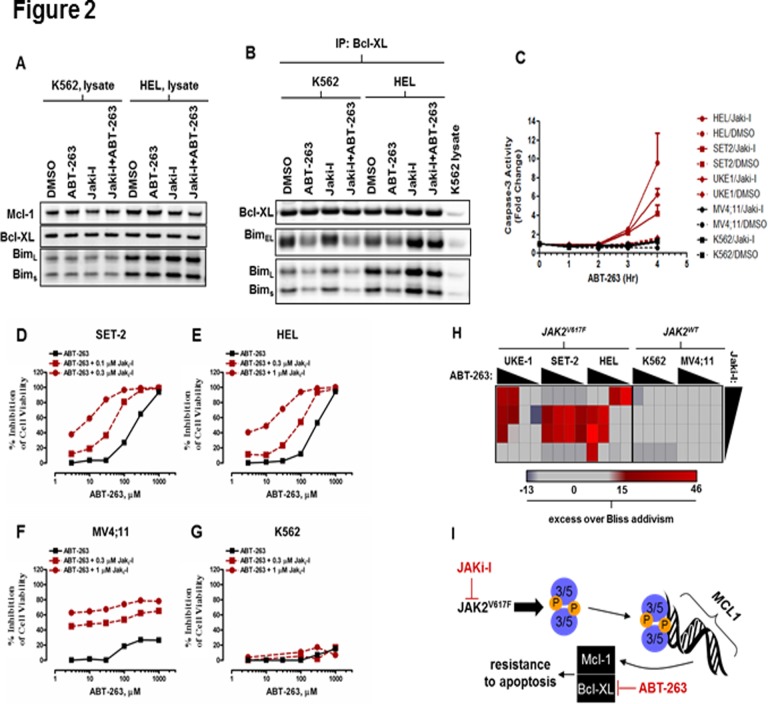
Combination of JAK2 and Bcl-2 family inhibitors yields synergistic antiproliferative activity in *JAK2* ^*V617F*^-harboring AML cell lines. (A/B) HEL and K562 cells were treated for 6 hr with 1 μM JAKi-I followed by 3 hr with 0.15 μM ABT-263, then lysates or Bcl-XL immunoprecipitates were prepared and immunoblotted. (C) Cells were treated for 6 hr with 1 μM JAKi-I followed by 0.15 μM ABT-263 over a 3-hr time period. Caspase-3 activity was determined at each time point. Data are from duplicate samples and are representative of at least three independent experiments. (D-G) Cells were treated in combination as indicated, and cell viability was determined after 72 hr. Data are means of duplicate determinations, and are representative of at least three independent experiments. (H) Drug-drug interactions were determined using a matrix of pairwise combinations covering half-log dose responses from 0.03 to 1 μM for both JAKi-I and ABT-263. Drugs were added simultaneously, and cell viability was determined after 72 hr. The data were then analyzed using the drug-drug interaction model of Bliss additivity^16^ to define dose combinations that were synergistic (values >15; red), antagonistic (values <-15; blue), or without effect (-15<values<15; gray). (I) Model of JAK2/Bcl-2 family inhibitor synergy. JAK2^V617F^ constitutively phosphorylates and activates STAT3/5, thus enforcing expression of the transcriptional target, Mcl-1. Mcl-1 collaborates with Bcl-XL to oppose apoptosis and support viability. Inhibition of JAK2 in this context silences JAK/STAT-driven transcription of Mcl-1, leaving survival largely dependent upon Bcl-XL. Neutralization of Bcl-XL with ABT-263 is then achieved at a lower dose and is sufficient to induce apoptosis.

Of the pro-apoptotic BH3-only proteins normally sequestered by anti-apoptotic members of the Bcl-2 family, Bim binds both Mcl-1 and Bcl-xL [17,18]. We therefore asked whether the loss of Mcl-1 induced by JAK inhibition resulted in increased binding of Bim to Bcl-xL. Although the abundance of total Bim protein was not altered following treatment with JAKi-I (Fig. 2A), Bim was enriched in Bcl-XL immunoprecipitates in the presence of the *JAK2* ^V617F^ mutation (Fig. 2B). In cells treated with ABT-263, Bim was displaced from Bcl-XL (Fig. 2B) irrespective of *JAK2* mutational status. To assess whether suppression of Mcl-1 by treatment with JAKi-I would indeed potentiate apoptosis induced by Bcl-xL/-2 inhibition, we pretreated cell lines with JAKi-I for 6 hr (time sufficient for Mcl-1 levels to decline) followed by ABT-263 and monitored the activity of caspase-3. Whereas neither JAKi-I nor ABT-263 alone induced caspase-3 activity, a synergistic induction was evident within four hours specifically in cell lines harboring *JAK2* ^V617F^ (Fig. 2C).

These data suggested that in JAK2-driven malignancies, the reduction in Mcl-1 that results from JAK/STAT inhibition could be leveraged in a therapeutic combination that simultaneously neutralizes Bcl-xL/-2. Only *JAK2* ^V617F^-positive AML lines were sensitized to ABT-263 upon JAK inhibition as indicated by the leftward shift in ABT-263 EC_50_ (Fig. 2D-G). We then assessed drug-drug interactions using a matrix of pairwise combinations that covered half-log dose-responses between 0.03 and 1 μM for both JAKi-I and ABT-263 and using 72-hr cell viability as an endpoint. The viability data were then analyzed using the Bliss additivity mode [19] to define dose combinations that were synergistic, antagonistic, or without effect. Synergistic interactions were observed for multiple dose combinations specifically in cell lines carrying the *JAK2* ^V617F^ lesion (Fig. 2H). Similar phenotypic enhancements by Ruxolitinib, a clinical relevant JAK inhibitor, combined with ABT-263 were also observed (data not shown). A recent study [20] also supported our data that Bcl-2/Bcl-xL inhibitor ABT-737 was effective in combination with JAK2 inhibition.

## Discussion

Targeting mutant JAK2 ^V617F^, which leads to constitutively activation of JAK2 and its downstream pathways, has potential as a therapeutic approach as that mutation leads to blockage of apoptosis and uncontrolled cellular proliferation.

Combination of JAK2 inhibitors with other therapeutic agents has demonstrated beneficial effects on growth inhibition of JAK2^V617F^-expressing cells. The combination of an Aurora kinase inhibitor (VX-680) with a JAK2 inhibitor (TG101209) has recently been shown to synergistically reduce the proliferation of JAK2^V617F^-positive cells. Also, the use of a JAK2 inhibitor in combination with suppression of the PI3K/Akt or mTOR pathways synergistically reduced the proliferation of JAK2^V617F^-positive cells [21]. Therefore, combinations that synergistically enhance efficacy provide the potential to reduce drug levels and reduce toxicity. In addition, combining two compounds with different mechanisms of action may reduce the probability of developing resistance to either of the drugs.

In this study, we expanded upon previous results [22,23] that the JAK inhibitor I impairs proliferation in *JAK2* mutant cell lines by demonstrating a key role of Mcl-1 regulation in this synergistic effect. Mcl-1 is apparently regulated by STAT3 as determined by CHIP analysis, which may also implicate STAT5 due to co-regulation by JAK. The biological properties of ABT-263, a potent, orally bioavailable, Bad-like, BH3 mimetic (Ki’s of <1 nmol/L for Bcl-2, Bcl-xL, and Bcl-w) have been reported previously [24]. In vivo, ABT-263 exhibited pronounced oral activity in multiple xenograft models, both as a single agent and in combination with standard of care chemotherapies [24]. In cells, ABT-263 inhibits the interaction between pro-apoptotic and anti-apoptotic Bcl-2 family proteins in both a mammalian two hybrid system and in FL5.12 cells. IL-3 withdrawal in FL5.12 cells has previously been shown to dramatically increase Bim and reduce Mcl-1 levels, resulting in the induction of apoptosis [25,26]. Recent studies indicated that Bcl-2 inhibitors, ABT-737 and ABT-199, do show synergy with imatinib in BCR-ABL cells [27,28].

The JAK/STAT pathway is constitutively activated (phosphorylated) in cells harboring the JAK^V617E^ mutation. As tyrosine phosphorylation of STAT proteins induces transcriptional activation through homodimerization, selective inhibition of STAT3/5 phosphorylation in *JAK2* ^V617F^-harboring leukemia lines suggested that transcriptional targets of STAT3/5 may be silenced selectively in these lines. Mcl-1 is a STAT transcriptional target [29,30,31] and was of particular interest as it has been shown to confer resistance to apoptosis following inhibition of Bcl-xL and Bcl-2 [10,12,13]. Mcl-1 expression is, therefore, transcriptionally enforced by the JAK/STAT pathway in AML cell lines harboring *JAK2* ^V617F^. This suggests that leukemias that express *JAK2* ^V617F^ may display a reduced threshold for apoptosis induced by ABT-263 in combination with JAKi-I. The presence of alternative STAT3/5 activating lesions in MV;411 (*FLT3* ^*ITD*^) and K562 (*BCR-ABL*), renders STAT3/5 phosphorylation JAK-independent [32,33,34]; therefore, resistant to the combination as demonstrated herein. The observation that ABT-263 fails to induce caspase-3 activity during this period indicates that the BH3-only proteins displaced from Bcl-xL/-2 are not sufficiently abundant to exceed the binding capacity of additional antiapoptotic members such as Mcl-1. These data indicate *JAK2* ^*V617F*^ constitutively phosphorylates and activates STAT3/5, thus enforcing expression of the transcriptional targets Mcl-1 and Bcl-xL. Mcl-1 collaborates with Bcl-xL to oppose apoptosis and support viability. Inhibition of JAK2 in this context silences JAK/STAT-driven transcription of Mcl-1, leaving survival largely dependent upon remaining Bcl-xL. Neutralization of Bcl-xL with ABT-263 is then achieved at a lower dose and is sufficient to induce apoptosis (Fig. 2I). These findings have broad implications for targeted combination therapy in JAK2-driven hematologic malignancies as well as MPN/MDS.

## Supporting Information

S1 Dataset JAKi-I was evaluated in a panel of 66 human protein kinases by TR-FRET enzyme assays as detailed in the Methods section, and Ki values determined. Individual Ki values are given in the table.
(XLS)

S2 Dataset Cells were treated for 6 hr with JAKi-I, and the abundance of Mcl-1 and Bcl-XL mRNA was determined by qPCR. Data represent means +/- standard deviation for two independent determinations each performed in triplicate (data in Summary tab). Individual experimental data in exp 051409 and repeat Mcl1 tabs.
(XLS)

S3 Dataset Quantitation of western blot data by LiCor Odyssey Imager. (XLS)

S4 Dataset HEL or K562 cells were transfected with either non-targeting (siNT-1) or Mcl-1-specific (siMcl1–1–4) siRNAs for 48 hr, subsequently treated for 72 hr with ABT-263, then lysates were prepared, and cell viability was determined. Data are means of duplicate samples and are representative of two independent experiments.
(XLS)

S5 Dataset The data are expressed as the “per cell” induction of Caspase-3/-7. In Fig. 2C the data are expressed as Caspase-3/7 activity divided by cell viability, and then this ratio is used to calculated the fold change comparing with control. This is a way to appropriately normalize the caspase induction to the cell number (which may change during treatment, *e*.*g*., cell number will be reduced as cell die).
(XLS)

S6 Dataset Cells were treated in combination as indicated, and cell viability was determined using alamarBlue after 72 hr. Data are means of duplicate determinations, and are representative of at least three independent experiments.
(XLS)
